# Persistent Organic Pollutants and Inflammatory Markers in a Cross-Sectional Study of Elderly Swedish People: The PIVUS Cohort

**DOI:** 10.1289/ehp.1307613

**Published:** 2014-05-23

**Authors:** Jitender Kumar, P. Monica Lind, Samira Salihovic, Bert van Bavel, Erik Ingelsson, Lars Lind

**Affiliations:** 1 Molecular Epidemiology and Science for Life Laboratory, and; 2 Occupational and Environmental Medicine, Department of Medical Sciences, Uppsala University, Uppsala, Sweden; 3 MTM Research Centre, School of Science and Technology, Örebro University, Örebro, Sweden; 4 Cardiovascular Epidemiology, Department of Medical Sciences, Uppsala University, Uppsala, Sweden

## Abstract

Background: Persistent organic pollutants (POPs) are compounds that are generated through various industrial activities and released in the surrounding environment. Different animal studies have shown effects of different POPs on various inflammatory markers.

Objective: Because very few studies have been conducted in humans, we assessed the associations between different POPs and inflammatory markers in a large population-based sample of elderly men and women (all 70 years of age) from Sweden.

Methods: This cross-sectional study investigated the concentrations of several polychlorinated biphenyls (PCBs), organochlorine pesticides, polychlorinated dibenzo-*p*-dioxin, and brominated diphenyl ether congeners and their association with a number of inflammatory markers [vascular cell adhesion molecule 1 (VCAM-1), intercellular adhesion molecule 1 (ICAM-1), E-selectin, C-reactive protein (CRP), total leucocyte count, tumor necrosis factor α (TNF-α), monocyte chemotactic protein 1 (MCP-1), and interleukin 6 (IL-6)] in 992 individuals. These individuals were recruited from the Prospective Investigation of the Vasculature in Uppsala Seniors (PIVUS) cohort. We used a total toxic equivalency (TEQ) value that measures toxicological effects with the relative potencies of various POPs.

Results: Following adjustment for potential confounders, the TEQ value (driven mainly by PCB-126) was significantly associated with levels of ICAM-1 (*p* < 10^–5^). A similar trend was also observed between sum of PCBs and VCAM-1 (*p* < 0.001). No significant associations were observed between levels of POPs and other inflammatory markers.

Conclusions: TEQ values were associated with levels of ICAM-1, to a lesser degree also with VCAM-1, but not with CRP and several other inflammatory markers. These findings suggest an activation of vascular adhesion molecules by POPs, and particularly by PCB-126.

Citation: Kumar J, Lind PM, Salihovic S, van Bavel B, Ingelsson E, Lind L. 2014. Persistent organic pollutants and inflammatory markers in a cross-sectional study of elderly Swedish people: The PIVUS cohort. Environ Health Perspect 122:977–983; http://dx.doi.org/10.1289/ehp.1307613

## Introduction

Persistent organic pollutants (POPs) are the organic compounds that are present in the surrounding environment and not easily degradable through environmental processes. Because of the industrial revolution in the past few centuries, a plethora of hazardous POPs generated directly or as by-products has been released into the environment. Once released, these chemicals persist for a long time and may reach concentrations that induce adverse health effects. Several of the chemical entities, including congeners of polychlorinated biphenyls (PCBs), organochlorine (OC) pesticides, polychlorinated dibenzo-*p*-dioxins, and brominated diphenyl ether (BDE) congeners, fall into this category. An overwhelming number of studies have shown that large majority of individuals born after the mid-20th century have been and still are exposed to POPs in everyday life (e.g., Hertsgaard 1996; Schecter et al. 2010). Bioaccumulation of POPs in the general population occurs primarily through ingestion of contaminated food (e.g., fish, meat, or dairy products) as well as via air and dust in indoor environments (Johnson et al. 2010; Letcher et al. 2010). Several cross-sectional and prospective studies have reported positive associations between levels of POPs and diseases such as obesity, atherosclerosis, diabetes, allergies, and cancers (Dirinck et al. 2011; Elobeid et al. 2010; Hardell et al. 2006a, 2006b; Lee et al. 2007, 2011, 2012a, 2012b; Lind and Lind 2012; Lind et al. 2012; Noakes et al. 2006; Persky et al. 2011, 2012; Ronn et al. 2011; Verner et al. 2008).

Some of the POPs, such as dioxins and PCBs, exhibit their biochemical and toxic effects through aryl hydrocarbon receptor (AhR)–mediated response (Beischlag et al. 2008; Hao and Whitelaw 2013). The AhR is a member of the family of basic helix–loop–helix transcription factors that are important for developmental processes and involved in regulation of biological responses to aromatic hydrocarbons by inducing the expression of target genes (Beischlag et al. 2008; Hao and Whitelaw 2013). The total toxic equivalency (TEQ) value using relative potencies of various POPs to bind to AhR has been developed to measure toxicological effects and risk characterization in different organisms (Van den Berg et al. 1998). The TEQ values can be calculated using toxic equivalency factors (TEFs) that express the toxicity of various POPs in relation to the most toxic dioxin, 2,3,7,8-tetrachlorodibenzo-*p*-dioxin (TEF = 1) (Van den Berg et al. 2006). TEFs are used for human health risk assessment for different POPs (Van den Berg et al. 1998). The applicability of the TEF approach for determining the toxicity of different PCB mixtures has been substantiated in both *in vivo* and *in vitro* studies (Bradlaw et al. 1980; Harris et al. 1993).

Experimental studies show that POPs can induce inflammation (Cheon et al. 2007; Hennig et al. 2002; Nishiumi et al. 2010; Peltier et al. 2013). Inflammation is a cluster of different responses to a trauma (e.g., exposure to toxic compounds) and may be initiated in different ways involving various pathways. Although inflammation plays an important role in the defense mechanism in biological systems, it may also lead to apparent damage in cases of severe response (Medzhitov 2008). Various toxic compounds may trigger an abnormal inflammatory response directly or indirectly through interfering with normal physiological functioning of cells or tissues (Medzhitov 2008). In a cross-sectional study of a large number of nondiabetic individuals, Kim et al. analyzed the influence of POP concentrations on inflammation and insulin resistance, showing the association of pesticides with increased levels of C-reactive protein (CRP) (Kim KS et al. 2012). Another study, on the impact of POPs on human adipose cells, showed that both precursor cells and adipocytes are targets of POPs and that these pollutants trigger mainly the inflammation pathway (Kim MJ et al. 2012). In a study from Japan involving 40 Yusho patients and 40 controls, Kawatsuka et al. (2013) demonstrated that serum levels of interleukin (IL)-17, IL-1β, IL-23, and tumor necrosis factor-α (TNF-α) were higher in patients who were exposed to POPs, including PCBs through consumption of contaminated rice (Kuwatsuka et al. 2013). Circulating inflammatory biomarkers such as CRP, IL-6, TNF-α, monocyte chemotactic protein 1 (MCP-1), intercellular adhesion molecule 1 (ICAM-1), vascular cell adhesion protein 1 (VCAM-1), and E-selectin have been associated with a variety of metabolic disorders and associated outcomes (Goldberg 2009).

Although several animal studies have been performed to show that POPs are related to inflammation, there are few data on humans, and in the existing studies only limited numbers of individuals have been included (Fang et al. 2012; Glynn et al. 2008; Hennig et al. 2002; Imbeault et al. 2012; Kim KS et al. 2012; Noakes et al. 2006; Sipka et al. 2008; Sipos et al. 2012; Weisglas-Kuperus et al. 1995). Therefore, we conducted this study using measurements of various circulating POPs in a large population-based sample of men and women 70 years of age from the Prospective Investigation of the Vasculature in Uppsala Seniors (PIVUS) cohort (http://www.medsci.uu.se/pivus/). In this cross-sectional study, our primary aim was to investigate the association of TEQ values derived from seven dioxin-like PCBs and octachlorodibenzo-*p*-dioxin (OCDD), the sum of OC pesticides, and the sum of PCBs with a variety of inflammatory markers [VCAM-1, ICAM-1, MCP-1, E-selectin, TNF-α, and IL-6, CRP, total leucocyte count (TLC)]. As an exploratory effort, our secondary aim was to analyze the associations between different individual POPs measured with these inflammatory markers.

## Materials and Methods

*Study participants*. All the participants in this study were 70-year-old individuals living in Uppsala, Sweden, and were chosen from the population register. A total of 1,016 individuals agreed to participate (participation rate, 50.1%) and provided their written informed consent. The Ethics Committee of Uppsala University approved the study.

All the study participants were asked to observe an overnight fast and not to use any medication or to smoke after midnight before being evaluated the next morning. Venous blood samples were drawn after participants’ overnight fast between 0800 and 1000 hours by one of three experienced nurses. Blood samples were centrifuged within 30 min, and plasma/serum were frozen within 1 hr and stored at –70°C until analysis. The temperature in the sampling room was kept constant at 22°C. A detailed questionnaire concerning smoking, medications, and medical history was filled in by participants (available from corresponding author on request). Standard laboratory techniques were employed to measure the fasting blood glucose as well as different lipid variables (Carlsson et al. 2009). A calibrated mercury sphygmomanometer (SD-43; Omron Healthcare, Myata, Japan) was used to measure blood pressure, and an average of three recordings was used. Kidney function was measured by calculating glomerular filtration rate following the Modification of Diet in Renal Disease formula (Levey et al. 2007). Details about recruitment of study participants and clinical characteristics have been published previously (Lind et al. 2005). Of 1,016 individuals, POPs could be measured in 992 participants only. Of these 992 individuals, 11.6% had diabetes, 1.1% were on lipid-lowering medication, and 10.6% were current smokers.

*Measurement of POPs*. POPs were measured in 992 participants from stored serum samples employing a slightly modified method by Sandau et al. (2003). The samples were kept in a –70°C freezer for approximately 5 years before analysis. The sampling, collection, and storage processes were performed in rooms kept free from POPs as far as possible. Details regarding measurements of different POPs can be found from a previous study by our group (Salihovic et al. 2012). Briefly, 1 mL formic acid was added to 0.5 mL plasma sample to denature proteins followed by sonication for 15 min. After 60 min, 1 mL of 3% isopropanol in water along with labeled ^13^C internal standards was added, and the mixture was sonicated again. Samples were loaded on a conditioned Oasis® HLB SPE (Waters, Milford, MA, USA) cartridge (6 cm^3^/150 mg) to perform solid-phase extraction. The cartridge was preconditioned with 3 mL of methanol followed by 3 mL of dicholoromethane, 6 mL of methanol/dichloromethane (1:1), 4.5 mL of methanol, and 4.5 mL of water. We used 3% isopropanol in water (6 mL) and 40% methanol in water (6 mL) to rinse the cartridge so as to remove all the proteins and interferences from the sorbent phase. After drying under vacuum and nitrogen for 40 min, the target compounds were eluted with 6 mL dicholoromethane/hexane (1:1) into 8 mL amber glass vials prespiked with 25 μL of N-tetradecane. Samples were then dried under nitrogen and reconstituted on hexane (500 μL). A small, activated multilayer silica gel column (2 mL, 1.5 g) was used for further cleanup, and 7.5 mL of hexane was passed through the column to achieve elution of analytes. After evaporation and addition of the ^13^C-labeled recovery standard, the final volume was adjusted to 25 μL *N*-tetradecane. A Micromass AutoSpec-Ultima (Waters) high-resolution gas chromatograph/high-resolution mass spectrometer was employed to perform the final measurements. We monitored the two most abundant ions of the chlorine or bromine cluster in addition to one ion for ^13^C-labeled internal and recovery standards by injecting 2 μL on a 6890N gas chromatograph (Agilent Technologies, Atlanta, GA, USA) containing a 30 m × 0.25 i.d. × 0.25 μm DB-5 capillary column (SGE Analytical Science, Victoria, Australia). The levels of POPs were normalized for the lipid content in plasma. To calculate the total amount of lipid present in each plasma sample, we used a summation formula based on the concentrations of serum cholesterol and triglyceride (Rylander et al. 2006).

*Quality control*. In each batch of 10 samples, quality control plasma samples and procedural blank samples were incorporated to ensure the quality. The blank samples were devoid of any target compounds at levels > 5% of the levels in samples except for *cis*-chlordane and *trans*-chlordane. Both chlordanes were present below the limit of detection (LOD) in 95% of the samples analyzed. The recovery of internal standards ranged from 60% to 110% and was satisfactory. The relative SD of 100 quality assurance/quality control (QA/QC) samples was < 25% for all the compounds measured except for one present at low levels and just above the LOD in the QA/QC sample. Further details of quality controls may be found elsewhere (Salihovic et al. 2012). Samples with POPs having concentration below the LOD were imputed and given LOD/2^–0.5^ values.

*TEQ value calculation*. TEQ values were calculated using seven mono- and non-*ortho*-substituted dioxin-like PCBs (PCBs 126, 169, 105, 118, 156, 157, 189) and OCDD as described by Van den Berg et al. (2006). The concentrations of PCBs and OCDD were multiplied by their respective TEF and then added. We also calculated the sum of concentrations of all PCBs measured and a separate sum of three OC pesticides.

*Measurement of inflammatory markers*. A total of eight inflammatory markers were used in this study. Serum high-sensitive CRP was measured by ultrasensitive particle enhanced immunoturbidimetric assay (Orion Diagnostica, Espoo, Finland) on a Konelab 20 autoanalyzer (Thermo Clinical Labsystems, Espoo, Finland). The interassay coefﬁcient of variation was 3.2%. Cytokines, chemokines, and adhesion molecules were analyzed on Evidence® array biochip analyzer (Randox Laboratories Ltd., Crumlin, UK). The method has been described previously by Fitzgerald et al. (2005). The functional sensitivities for different inﬂammatory markers measured were as follows: CRP, 0.1 mg/L; TLC, 0.2; IL-6, 0.3 pg/mL; TNF-α, 1.8 pg/mL; MCP-1, 19.4 pg/mL; ICAM-1, 18.6 pg/mL; VCAM-1, 3.1 pg/mL; and E-selectin, 3.1 pg/mL.

*Statistical analysis*. Variables were evaluated for non-normality, and variables showing skewed distribution were log transformed to achieve normal distribution. We used linear regression to analyze the association of TEQ values, sum of PCBs, or sum of OC pesticides (independent variables) with different inflammatory markers (dependent variables). A variety of covariates including sex, education (three discrete levels), physical activity (four different groups), waist circumference (centimeters), smoking (current smokers, previous smokers, and nonsmokers), kidney function (glomerular filtration rate), fasting blood glucose, systolic blood pressure, body mass index (BMI), lipid profile (levels of high- and low-density lipoproteins cholesterol, triglycerides) were considered and adjusted for. Two different statistical models were employed in regression analysis: model A, considering sex and kidney function as covariates; and model B, considering all covariates. Additionally, a squared term of POPs was included in the models to evaluate potential nonlinear relationships. Interactions between levels of POPs and sex for POPs and all outcomes were evaluated by introducing an interaction term between POP and sex together with the POP and sex terms. In the primary analysis, TEQ values, sum of PCBs, and sum of OC pesticides were analyzed for their association with different inflammatory markers studied. The alpha threshold was set by dividing 0.05 by the number of inflammatory markers analyzed (i.e., a Bonferroni correction; 0.05/8 = 0.0063). Further analysis was performed in a subsample of presumably healthier individuals (nonsmokers and nondiabetic and nonhyperlipidemic individuals) to confirm the findings from primary analysis. Additionally, participants were divided into two groups based on median BMI, and the significant findings were confirmed. Smoking was also analyzed in a similar manner. Because the studied population is older, we also investigated whether there is an impact of certain medications [aspirin, cortisone, and nonsteroidal anti-inflammatory drugs (NSAIDs)] on significant outcomes. In the secondary exploratory analysis, levels of individual POPs were examined for their association with inflammatory markers. No Bonferroni adjustment was made in secondary analysis, but the *p*-value < 0.0063 was considered significant. The statistical software package STATA (version 12; StataCorp, College Station, TX, USA) was employed to perform all statistical analyses.

## Results

The general/clinical characteristics of the individuals recruited in this study are shown in Table 1. There were an almost equal percentage of males and females in the study group. Sixty-seven percent of the individuals were overweight (BMI ≥ 25.0 kg/m^2^), and 22% of the individuals were obese (BMI ≥ 30.0 kg/m^2^). Two of the OC pesticides (*trans*-chlordane and *cis*-chlordane) were not detectable in > 30% of the study population and hence were not analyzed further. A total of 21 POPs involving 16 PCBs, 3 OC pesticides, 1 BDE, and 1 OCDD were detected in > 70% of individuals and were taken up further for analysis (Table 2). Distribution of all 21 POPs in the studied population is shown in Table 2. Median (interquartile range) of TEQ values was calculated using eight POPs (7 PCBs and 1 OCDD) and found to be 9.8 (6.7–13.9). Logarithmic transformation was performed on these values to achieve normal distribution (Table 2).

**Table 1:** General/clinical characteristics and inflammatory markers of the studied individuals.

Variable	*n*	Median (IQR)	Min–Max
BMI (kg/m^2^)	991	26.6 (24.0–29.6)	16.6–49.8
WC (cm)	980	90 (84–98)	60–134
Glucose (mmol/L)^*a*^	988	5.0 (4.6–5.4)	2.8–19.9
SBP (mmHg)	987	148 (134–164)	84–230
LDL (mmol/L)	986	3.3 (2.8–3.9)	0.8–6.9
HDL (mmol/L)	988	1.4 (1.2–1.8)	0.6–3.8
TG (mmol/L)^*a*^	988	1.15 (0.87–1.51)	0.35–4.8
GFR (mL/min/1.73m^2^)^*a*^	987	78.9 (65.8–94.9)	23.2–210.8
ICAM-1 (mg/L)	991	226 (193–266)	88–886
VCAM-1 (mg/L)	991	520 (457–601)	217–1661
E-Selectin (mg/L)^*a*^	991	14.8 (11.1–18.9)	3.5–98.8
IL-6 (pg/mL)^*a*^	974	4.2 (2.2–15.0)	0.3–800
TNF-α (pg/mL)^*a*^	987	3.7 (2.9–4.9)	1.2–183.2
MCP-1 (pg/mL)	982	382 (308–467)	16–973
CRP (mg/L)^*a*^	991	1.20 (0.62–2.32)	0.18–93.46
TLC (× 10^9^ cells/L)	986	5.5 (4.7–6.5)	1.7–15.2
Abbreviations: CRP, C-reactive protein; GFR, glomerular filtration rate; HDL, high-density lipoprotein; ICAM-1, inter­cellular adhesion molecule 1; IL-6, interleukin 6; IQR, interquartile range; LDL, low-density lipoprotein; Max, maximum; MCP-1, monocyte chemotactic protein-1; Min, minimum; SBP, systolic blood pressure; TG, triglyceride; TLC, total leucocyte count; TNF-α, tumor necrosis factor α; VCAM-1, vascular cell adhesion protein 1; WC, waist circumference. ^***a***^Log-transformed values are reported.			

**Table 2:** Distribution of POPs studied along with their summary measures.

POP	*n*	GM^*a*^	Median (IQR)^*a*^	Median (IQR)^*b*^	Min–Max^*b*^	% < LOD
PCB-74	991	88.4	91.4 (63.8–128.2)	2.7 (2.4–3.0)	0.4–4.1	0
PCB-99	991	87.8	90.7 (62.4–132.0)	2.7 (2.3–3.0)	0.2–4.9	0.5
PCB-105	991	31.6	32.0 (21.0–46.8)	1.6 (1.3–2.0)	–0.3–4.0	0
PCB-118	990	194.5	200.6 (136.4–281.0)	3.5 (3.1–3.8)	1.5–5.5	0
PCB-126	985	38.4	40.4 (21.6–71.8)	1.9 (1.3–2.4)	–2.3–4.1	4.5
PCB-138	991	807.0	819 (619–1115)	4.9 (4.6–5.2)	2.6–6.0	0.4
PCB-153	991	1394.0	1428 (1114–1846)	5.5 (5.2–5.7)	2.9–6.5	0
PCB-156	991	151.5	154.2 (118.6–197.6)	3.2 (3.0–3.5)	0.8–4.3	0
PCB-157	991	28.0	28.0 (21.4–37.0)	1.5 (1.2–1.8)	–0.2–3.6	0
PCB-169	985	166.4	171.4 (130.6–219.8)	3.1 (2.8–3.3)	–0.3–4.5	0.3
PCB-170	991	489.0	497.2 (385.4–632.8)	4.4 (4.2–4.6)	2.3–5.8	0
PCB-180	991	1147.0	1165 (917–1487)	5.2 (5.0–5.5)	3.2–6.4	0
PCB-189	991	20.2	19.2 (14.6–25.8)	1.1 (0.9–1.4)	–1.1–5.0	0
PCB-194	991	101.9	119.4 (87.6–158.8)	3.0 (2.7–3.2)	–0.8–4.4	1.4
PCB-206	991	26.2	26.8 (20.8–35.2)	1.5 (1.2–1.8)	–1.1–3.3	0
PCB-209	991	25.0	26.2 (19.6–34.6)	1.5 (1.2–1.7)	–1.3–2.9	0
OCDD	986	2.7	2.6 (1.4–4.1)	–0.9 (–1.4–0.4)	–2.1–1.1	19.4
HCB	991	255.5	254.0 (189.2–336.6)	3.7 (3.4–4.0)	2.6–6.4	1.4
*p,p’*-DDE	991	1847.0	1,858 (1,024–3,451)	5.7 (5.1–6.3)	0.7–8.4	0
BDE-47	991	15.1	12.6 (9.0–19.4)	0.7 (0.5–1.1)	–0.2–6.2	27.8
TNC	991	137.3	139.2 (91.6–211.2)	3.1 (2.7–3.5)	0.8–4.9	0
TEQ	979	9.6	9.8 (6.7–13.9)	0.5 (0.1–0.8)	–2.1–1.9	—
Sum PCBs	984	4908.0	4,988 (3,942–6,300)	47.3 (43.3–50.9)	16.6–62.5	—
Sum OCP	991	2402.0	2,298 (1,475–3,945)	12.6 (11.6–13.5)	4.8–17.4	—
Abbreviations: BDE, bromodiphenyl ether; *p,p’*-DDE, dichlorodiphenyldichloroethylene; GM, geometric mean; HCB, hexachlorobenzene; IQR, interquartile range; LOD, limit of detection; Max, maximum; Min, minimum; OCDD, octachloro­dibenzo-*p*-dioxin; OCP, organochlorine pesticide; PCB, polychlorinated biphenyls; TEQ, total equivalency value; TNC, *trans*-nonachlordane. ^***a***^Actual concentrations of pollutants (pg/mL) in the plasma. ^***b***^Lipid-normalized and log-transformed concentrations of POPs (ng/g of lipid).						

*Association of TEQ value, sum of PCBs, and sum of OC pesticide concentrations with cell adhesion molecules*. The levels of different cell adhesion molecules ICAM-1, VCAM-1, and E-selectin were analyzed for their association with the TEQ values using two different statistical models in linear regression. In model A, the TEQ value was found to be significantly associated with levels of ICAM-1 in a positive manner (*p* = 2.7 × 10^–5^). In model B, the significance level did not change much and remained highly significant (*p* = 3.6 × 10^–5^) (Table 3). When the concentrations of individual PCBs were analyzed for their association with levels of ICAM-1, mainly PCB-126 showed significant association in both models A and B (*p* < 10^–8^; see also Supplemental Material, Tables S1 and S2).

**Table 3:** Associations [β (95% CI)] of TEQ values, sum of PCBs, and sum of OC pesticide concentrations with inflammatory markers studied.

Marker	TEQ				Sum of PCBs				Sum of OC pesticides			
	Model A	*p*-Value	Model B	*p*-Value	Model A	*p*-Value	Model B	*p*-Value	Model A	*p*-Value	Model B	*p*-Value
ICAM-1	16.35 (8.76, 23.94)	2.7 × 10^–5^	16.36 (8.64, 24.08)	3.6 × 10^–5^	0.05 (–0.59, 0.68)	0.89	0.11 (–0.55, 0.76)	0.75	0.67 (–1.98, 3.32)	0.62	–0.17 (–2.93, 2.59)	0.91
VCAM-1	25.26 (7.40, 43.11)	0.006	26.06 (7.85, 44.27)	0.005	2.25 (0.76, 3.73)	0.003	2.55 (1.02, 4.07)	0.001	9.20 (3.017, 15.37)	0.004	4.50 (–1.98, 10.99)	0.17
E-Selectin	–0.02 (–0.07, 0.03)	0.44	–0.01 (–0.06, 0.04)	0.69	–0.002 (–0.01, 0.002)	0.36	–0.001 (–0.01, 0.003)	0.55	0.02 (–0.002, 0.03)	0.07	–0.003 (–0.02, 0.02)	0.76
IL-6	0.02 (–0.18, 0.22)	0.82	0.05 (–0.16, 0.26)	0.62	0.01 (–0.01, 0.02)	0.43	0.01 (–0.01, 0.03)	0.41	0.01 (–0.06, 0.08)	0.76	0.004 (–0.07, 0.08)	0.92
TNF-α	0.01 (–0.06, 0.09)	0.77	0.01 (–0.07, 0.09)	0.78	–0.003 (–0.01, 0.004)	0.41	–0.003 (–0.01, 0.004)	0.37	–0.01 (–0.03, 0.02)	0.62	–0.01 (–0.04, 0.02)	0.35
MCP-1	3.74 (–13.47, 20.95)	0.67	2.96 (–14.78, 20.70)	0.74	–0.32 (–1.76, 1.11)	0.66	–0.29 (–1.78, 1.20)	0.7	1.11 (–4.87, 7.10)	0.72	–1.42 (–7.73, 4.89)	0.66
CRP	–0.04 (–0.16, 0.09)	0.57	0.06 (–0.07, 0.18)	0.36	–0.01 (–0.02, –0.001)	0.03	–0.002 (–0.01, 0.01)	0.7	–0.004 (–0.05, 0.04)	0.86	–0.05 (–0.09, –0.01)	0.03
TLC	0.11 (–0.08, 0.30)	0.24	0.09 (–0.09, 0.27)	0.33	–0.01 (–0.03, 0.003)	0.11	–0.01 (–0.02, 0.01)	0.26	0.06 (–0.004, 0.13)	0.06	0.004 (–0.06, 0.07)	0.91
Abbreviations: CRP: C-reactive protein; ICAM-1: intercellular adhesion molecule 1; IL-6: interleukin 6; MCP-1: monocyte chemotactic protein-1; OC: organochlorine; PCB: polychlorinated biphenyls; TLC: total leucocyte count.; TNF-α: tumor necrosis factor alpha; VCAM-1: vascular cell adhesion protein 1. Model A: linear regression model adjusted for sex and kidney function; model B: linear regression model adjusted for sex, kidney function, smoking, BMI, waist circumference, blood glucose, systolic blood pressure, high-density lipoprotein, low-density lipoprotein, triglycerides, exercise habits, and education.												

When the TEQ values were analyzed for their association with levels of VCAM-1, both models A and B showed results similar to those of ICAM-1 but were less significant (*p* = 0.006 and 0.005, respectively, Table 3). In this case, the association was driven by a number of PCBs (*p* = 0.001 for sum of PCBs), and not only PCB-126 (see Supplemental Material, Table S2). The sum of OC pesticides showed significant positive association with VCAM-1 in model A (*p* = 0.004, Table 3) but not in model B (*p =* 0.17, Table 4).

**Table 4:** Association [β (95% CI)] of individual OC pesticides with inflammatory markers^*a*^ studied.

Marker	HCB	*p*-Value	TNC	*p*-Value	*p,p’*-DDE	*p*-Value
ICAM-1	–11.83 (–21.32, –2.34)	0.02	–7.251 (–14.19, –0.31)	0.04	3.69 (–0.65, 8.03)	0.10
VCAM-1	2.19 (–19.93, 24.32)	0.85	5.10 (–11.12, 21.33)	0.54	7.12 (–3.01, 17.24)	0.17
E-Selectin	–0.022 (–0.08, 0.04)	0.50	–0.008 (–0.05, 0.04)	0.75	0.001 (–0.03, 0.03)	0.95
IL-6	0.21 (–0.05, 0.46)	0.11	–0.04 (–0.23, 0.15)	0.67	–0.01 (–0.13, 0.10)	0.81
TNF-α	–0.03 (–0.12, 0.07)	0.55	–0.005 (–0.07, 0.07)	0.89	–0.02 (–0.07, 0.02)	0.28
CRP	–0.02 (–0.17, 0.13)	0.79	–0.09 (–0.20, 0.02)	0.11	–0.083 (–0.15, –0.02)	0.016
MCP-1	0.78 (–20.92, 22.48)	0.94	–11.35 (–27.29, 4.6)	0.16	0.69 (–9.15, 10.53)	0.89
TLC	–0.014 (–0.23, 0.2)	0.9	–0.14 (–0.30, 0.02)	0.09	0.05 (–0.05, 0.15)	0.29
Abbreviations: CRP, C-reactive protein; *p,p*’-DDE, dichlorodiphenyldichloroethylene; HCB, hexachlorobenzene; ICAM-1, intercellular adhesion molecule 1; IL-6, interleukin 6; MCP-1, monocyte chemotactic protein-1; TLC, total leucocyte count. TNC, *trans*-nonachlordane; TNF-α, tumor necrosis factor α; VCAM-1, vascular cell adhesion protein 1. ^***a***^Linear regression model adjusted for sex, kidney function, smoking, BMI, waist circumference, blood glucose, systolic blood pressure, high-density lipoprotein, low-density lipoprotein, triglycerides, exercise habits, and education.						

No significant association between TEQ value or sum of PCBs and levels of E-selectin was observed in our study (Table 3). Further, no significant association for sum of OC pesticides was observed in model B with levels of cell adhesion molecules studied (Table 3). When individual POPs were analyzed, PCB-209 showed a significant association with levels of E-selectin in model A, but this significance was attenuated when adjusted for additional covariates in model B (see Supplemental Material, Tables S1 and S2).

*Association with downstream inflammation indicators*. When the TEQ value, sum of OC pesticides, and sum of PCBs were analyzed for their association with downstream inflammatory indicators (CRP and TLC) in both models A and B, no significance was observed (Table 3). Although levels of some of the individual PCBs (156, 157, 170, 180, 206, 209) were found to be significantly associated with CRP in model A (*p* < 0.001), the significance disappeared when model B, including a large number of covariates, was employed (see Supplemental Material, Tables S1 and S2). Further, PCB-126 and *p,p,´*-DDE (dichlorodiphenyldichloroethylene) showed significant association with TLC (*p* = 0.006 and 0.003, respectively) but only in model A (see Supplemental Material, Tables S1 and S2).

*Association with cytokines*. In linear regression analysis, no statistical significance was observed when TEQ values, sum of PCBs, or sum of OC pesticides were analyzed for their association with levels of different cytokines (IL-6, TNF-α, and MCP-1) in models A and B (Table 3). Further, similar nonsignificant results were observed with individual POPs (see Supplemental Material, Tables S1 and S2).

*Additional analysis*. Because a large proportion of individuals studied were either overweight or obese in the studied cohort, we divided these individuals into two groups based on median BMI (26.6 kg/m^2^) and compared the association of TEQ and PCB-126 on ICAM-1 between these groups. The association observed for TEQ values was stronger in the group that had higher median BMI compared with others (see Supplemental Material, Table S3). Further, TEQ value and PCB-126 showed significant association with levels of ICAM-1 only in nonsmokers (see Supplemental Material, Table S3).

Additionally, to investigate the influence of medications on significant findings, we excluded individuals on medications in a stepwise manner (individuals taking aspirin, cortisone, or NSAIDs at each step). Association of TEQ values, sum of PCBs, and sum of OC pesticides with ICAM-1 or VCAM-1 was similar in all three groups (see Supplemental Material, Table S4).

To understand association of confounders with inflammatory markers as well as POP exposures, we performed univariate associations (see Supplemental Material, Table S5–S8).

No consistent nonlinear effects or sex–POP interactions were observed (data not shown).

*Association in subsample*. To exclude the impact of smoking, diabetes, and hyperlipidemia, we extracted a subsample of presumably healthy individuals who were nonsmokers, nondiabetic, and had a normal lipid profile (*n* = 776). When the association analysis was performed between POPs and inflammatory markers in this subsample, significant associations observed did not differ from those of the total samples (data not shown).

## Discussion

In the present study, we report the associations of TEQ values, sum of PCBs, and sum of OC pesticides with a number of inflammatory markers. While analyzing the association of TEQ values (representing concentration of seven dioxin-like PCBs and OCDD) with cell adhesion molecules that are considered to be classical inflammatory mediators, we found significant association mainly with two adhesion molecules, ICAM-1 and VCAM-1. Both of these molecules belong to immunoglobulin supergene family mediating attachment of leukocytes to vascular endothelium and their trans-endothelial migration, thereby having an important inﬂuence on inﬂammatory reactions (Muller 2009).

*Cell adhesion molecules*. Several experimental studies have shown potent vascular effects of different PCBs and their interaction with endothelial cells through the inflammatory response (e.g., Hennig et al. 2002). Studies performed in porcine endothelial cells to analyze the impact of coplanar PCBs (e.g., PCBs 77, 126, or 169) have shown that they have a concentration-dependent oxidative stress response and subsequent proinflammatory events (Hennig et al. 1999, 2002). When cultured endothelial cells are exposed to individual PCBs (PCBs 77, 126, and 169) with different concentrations (0.5, 1.0, and 2.5 μM), concentration-dependent increases in cellular oxidative stress have been observed compared with the control media (Hennig et al. 2002). When incubated with PCBs, these cells have shown increased production of IL-6. Further, the levels of VCAM-1 mRNA in endothelial cells treated with PCB-77 have been found to be significantly higher compared to control culture (Hennig et al. 2002). The most common route of PCB exposure in humans is through food chain (Agency for Toxic Substances and Disease Registry 2000). Sipka et al. (2008) have used an *in vivo* mouse model to show that orally administered PCBs mediated inflammatory mediators. Oral administrations of individual PCBs (150 μmol/kg body weight by oral gavage) have been found to activate a variety of specific inflammatory mediators. The mRNA levels of both ICAM-1 and VCAM-1 have been found to be significantly increased in organs such as the liver, lungs, and brain in response to the exposure to different PCBs (Sipka et al. 2008). This increase in mRNA expression is both time and dose dependent. Another study analyzing the impact of PCB-118 (oral gavage with 150 μmol/kg body weight) on formation of brain metastases has shown increased levels of VCAM-1 mRNA levels when mice were exposed to PCB-118 (Sipos et al. 2012).

Both ICAM-1 and VCAM-1 mediate adhesion-dependent cell-to-cell interactions in the vascular system and have an important inﬂuence on inﬂammatory reactions (Muller 2009). They play an important role in the initial phase of pathogenesis of atherosclerosis through migration of leukocytes and their adherence to the endothelium—one of the initial steps in pathogenesis of atherosclerosis (Cybulsky and Gimbrone 1991). The present findings are therefore in accordance with recently published data from the PIVUS study, where we found TEQ and PCBs to be related to carotid artery atherosclerosis measured by ultrasound (Lind et al. 2012). The toxicity due to coplanar PCB exposure, which binds to AhR with high affinity, has been well documented. However, recent studies have also suggested that non-coplanar PCBs may also produce adverse effects. Several non-coplanar PCBs (PCBs 170, 180, 206, 209) have shown positive association with inflammatory response via VCAM-1. Although how non-coplanar PCBs exert their effects remains unclear, it may be possible that these non-coplanar PCBs may bind to not yet identified PCB receptor(s).

*Downstream inflammation indicators*. In a cross-sectional study including the non-diabetic individuals, Kim et al. have analyzed the association of levels of various serum POPs with CRP (Kim KS et al. 2012). In that study, two different statistical models (including a number of covariates) were considered for analysis. Kim et al. observed that OC pesticides follow a significant positive trend with levels of CRP, but the association turns insignificant when additional adjustments for waist circumference and BMI are made. In the present study, we made similar observations with regard to some of the PCBs. Although a few individual PCBs showed significant associations with levels of CRP in model A, the association was attenuated to nonsignificance in model B. We did not observe any significant association between TEQ values, sum of PCBs, or sum of OC pesticides with CRP in our cohort.

When we analyzed total leucocyte counts for their association with different TEQ values, sum of PCBs, or sum of OC pesticides, we saw no significant association. A few studies, mainly in infants, have looked at POPs exposure and their association with total leucocyte counts (Glynn et al. 2008; Weisglas-Kuperus et al. 1995). Glynn et al. (2008) performed a study in infants from a location similar to ours showing that infants with greater exposure to different POPs have significantly higher mean numbers of total WBCs than infants in the reference category with the lowest exposure. Although two of the individual POPs (PCBs 126 and 209) were significantly associated with TLC in our study, these POPs became insignificant when adjusted for all the covariates in model B. The difference in age between our PIVUS participants and the infants from the study by Glynn et al. (2008), along with the respiratory infections experienced by these infants, may account for the differences in the observations. Another study in Dutch infants did not find any significant differences in total leucocyte counts due to the exposure to different POPs (Weisglas-Kuperus et al. 1995).

*Cytokines*. Only a very few studies have looked at the effects of POPs on the levels of cytokines in humans (Imbeault et al. 2012; Kim MJ et al. 2012). In a study including 109 individuals from Canada, where the association of elevated levels of POPs with activation of immune response was studied, a weak but significant association was observed (Imbeault et al. 2012). We observed no association between levels of POPs and proinflammatory cytokines (IL-6, MCP-1, and TNF-α). Differences in results between two studies may be attributable to various factors. The presence of other diseases (e.g., arthritis and cardiovascular and chronic respiratory diseases) experienced by these Canadian people may enhance the levels of various cytokines. Further, the number of individuals recruited in the Canadian study was small.

Studies have shown positive correlations between concentrations of different POPs with different measures of adiposity, such as BMI, fat mass, or waist circumference (Kim MJ et al. 2011; Pelletier et al. 2003; Porta et al. 2010; Roos et al. 2013). The effect size of POPs on significant inflammatory markers observed in our study was more than double in individuals with BMI above median than with those below the median. Because increased adiposity leads to higher burden of POPs that may augment the inflammatory response (La Merrill et al. 2013), higher effect size of POPs in individuals with BMI above median was in the expected direction. In a recent study, Baker et al. (2013) showed that mice treated with PCBs (doses of 2.5–248 mg/kg for PCB-77 and 0.3–3.3 mg/kg for PCB-126 by oral gavage) have increased glucose and insulin tolerance due to the increased accumulation of PCBs in adipose tissue, thereby resulting in higher expression of inflammatory markers such as TNF-α.

It has been hypothesized that consuming fishes or fish oil may have beneficial health effects. On one hand, we may see favorable impact on health due to such nutrients as omega-3 fatty acids that are present in fish and may control inflammation. However, these fishes may be high in POPs concentrations that may negate the positive outcomes (Turunen et al. 2013). Although we do not have such data in our cohort, the results observed in our study may be influenced by diet and nutrients consumed (especially fish and fish oil) by the participants.

*Strengths and limitations*. The main strength of this study is the inclusion of a large number of POPs and a variety of inflammatory markers. The study was conducted in a large community-based sample from the general population. A large number of POPs as well as inflammatory markers were analyzed.

Because the study was conducted in a cohort of elderly Caucasian individuals only, caution is needed in generalizing the results to individuals of other age groups or ethnicities. Individuals were recruited from a limited geographical location, thereby limiting the extrapolation of results to other locations. The associations for individual POPs shown in the Supplemental Material were not corrected for multiple test corrections and therefore should be treated with caution. The TEF/TEQ approach may overestimate the inflammatory response of various POPs studied because other agents may cause inflammation. There are natural dietary components present as well that are formed during cooking and mimic the AhR agonists, thereby augmenting inflammatory response, but these are not accounted for in the estimation of TEQ exposure (Denison and Nagy 2003).

## Conclusion

The TEQ values were found to be associated with levels of ICAM-1, and to a lesser degree to VCAM-1, but not to CRP and several other inflammatory markers. These findings suggest an activation of vascular adhesion molecules by POPs and particularly by PCB-126. These findings might partly explain why high levels of POPs are related to atherosclerosis and other disorders in which vascular inflammation plays an important part.

## Supplemental Material

(368 KB) PDF
