# Supplementary material for: Persistent Organic Pollutants and Inflammatory Markers in a Cross-Sectional Study of Elderly Swedish People: The PIVUS Cohort
Source: Environ Health Perspect. 2014 May 23;122(9):977–83. doi: 10.1289/ehp.1307613 (PMC4154217; doi:10.1289/ehp.1307613)
Supplement: (368 KB) PDF [file ehp.1307613.s001.pdf]

Environmental Health Perspectives doi:10.1289/ehp.1307613

Supplemental Material

Persistent Organic Pollutants and Inflammatory Markers in a Cross-Sectional Study of Elderly Swedish People: The PIVUS Cohort

Jitender Kumar, P. Monica Lind, Samira Salihovic, Bert van Bavel, Erik Ingelsson, and Lars Lind

Table of Contents **Page**

Table S1. Association [β (95% CI)] of individual POPs with inflammatory markers in model A **2**

Table S2. Association [β (95% CI)] of individual POPs with inflammatory markers in model B **3**

Table S3. Association [β (95% CI)] of TEQ and PCB-126 with ICAM-1 into different groups based on median BMI or smoking status **4**

Table S4. Association [β (95% CI)] of summary measures of POPs and medication with inflammatory markers studied **5**

Table S5. Association [β (95% CI)] of all the confounders with inflammatory markers studied **6**

Table S6. Association [β (95% CI)] of all the confounders with summary measures of POPs, pesticides and dioxin studied **7**

Table S7. Association [β (95% CI)] of all the confounders with PCBs (PCB-74 to PCB-156) studied **8**

Table S8. Association [β (95% CI)] of all the confounders with PCBs (PCB-157 to PCB-209) studied **9**

Table S1. Association [β (95% CI)] of individual POPs with inflammatory markers in model A.

POP	ICAM-1	p-value	VCAM-1	p-value	E-selectin	p-value	IL-6	p-value	TNF-α	p-value	MCP-1	p-value	CRP	p-value	TLC	p-value
PCB-74	-6.52 (-14.15, 1.12)	0.10	18.93 (1.17, 36.69)	0.037	0.02 (-0.03, 0.07)	0.48	0.09 (-0.11, 0.29)	0.36	0.002 (-0.07, 0.08)	0.97	-5.57 (-22.66, 11.53)	0.52	-0.03 (-0.15, 0.09)	0.65	-0.20 (-0.39, -0.02)	0.03
PCB-99	-0.45 (-6.99, 6.09)	0.89	11.57 (-3.63, 26.76)	0.14	0.03 (-0.02, 0.07)	0.21	0.14 (-0.03, 0.31)	0.10	-0.004 (-0.07, 0.06)	0.89	-8.86 (-23.46, 5.74)	0.24	-0.007 (-0.11, 0.10)	0.89	-0.08 (-0.24, 0.08)	0.34
PCB-105	-1.95 (-8.43, 4.54)	0.56	20.42 (5.38, 35.46)	0.008	0.047 (0.004, 0.09)	0.032	0.003 (-0.16, 0.17)	0.97	0.01 (-0.05, 0.08)	0.71	-3.63 (-18.12, 10.87)	0.62	0.008 (-0.10, 0.11)	0.87	-0.189 (-0.35, -0.03)	0.02
PCB-118	-2.13 (-9.34, 5.09)	0.56	29.15 (12.44, 45.86)	0.0007	0.04 (-0.01, 0.09)	0.08	0.09 (-0.09, 0.28)	0.33	0.01 (-0.06, 0.08)	0.77	-4.86 (-21.0, 11.28)	0.56	0.01 (-0.11, 0.13)	0.89	-0.21 (-0.39, -0.03)	0.02
PCB-126	12.75 (8.43, 17.08)	$1.9*10^{-9}$	15.05 (4.87, 25.24)	0.004	0.004 (-0.03, 0.03)	0.80	-0.02 (-0.13, 0.09)	0.71	0.01 (-0.03, 0.06)	0.54	0.53 (-9.55, 10.62)	0.92	0.04 (-0.04, 0.11)	0.33	0.15 (0.04, 0.26)	0.006
PCB-138	2.96 (-5.41, 11.33)	0.49	23.76 (4.34, 43.19)	0.017	0.01 (-0.04, 0.07)	0.64	0.18 (-0.03, 0.40)	0.10	-0.02 (-0.11, 0.06)	0.57	-3.54 (-22.26, 15.18)	0.71	-0.07 (-0.21, 0.06)	0.30	0.042 (-0.17, 0.25)	0.69
PCB-153	0.84 (-8.32, 10.01)	0.86	25.38 (4.12, 46.65)	0.02	-0.003 (-0.06, 0.06)	0.94	0.16 (-0.08, 0.39)	0.20	-0.03 (-0.12, 0.06)	0.46	-8.16 (-28.63, 12.31)	0.44	-0.16 (-0.30, -0.01)	0.04	-0.06 (-0.29, 0.16)	0.58
PCB-156	-9.82 (-19.28, -0.37)	0.04	14.91 (-7.12, 36.94)	0.19	-0.06 (-0.12, 0.01)	0.08	0.11 (-0.14, 0.35)	0.40	-0.02 (-0.11, 0.07)	0.63	10.02 (-11.19, 31.23)	0.36	-0.23 (-0.38, -0.08)	0.003	-0.14 (-0.38, 0.09)	0.22
PCB-157	-7.32 (-15.86, 1.22)	0.09	9.83 (-10.04, 29.70)	0.33	-0.07 (-0.12, -0.01)	0.02	0.02 (-0.20, 0.25)	0.84	-0.02 (-0.10, 0.06)	0.65	-7.60 (-26.75, 11.56)	0.47	-0.22 (-0.35, -0.08)	0.002	-0.10 (-0.31, 0.11)	0.34
PCB-169	3.82 (-4.87, 12.51)	0.39	32.80 (12.65, 52.95)	0.0015	-0.06 (-0.12, -0.01)	0.03	0.15 (-0.07, 0.37)	0.18	-0.01 (-0.10, 0.07)	0.74	4.90 (-14.56, 24.37)	0.62	-0.18 (-0.32, -0.04)	0.01	-0.07 (-0.28, 0.15)	0.53
PCB-170	-2.06 (-12.22, 8.10)	0.69	24.11 (.514, 47.71)	0.05	-0.06 (-0.13, 0.01)	0.09	0.14 (-0.13, 0.40)	0.31	-0.04 (-0.14, 0.06)	0.40	1.70 (-21.05, 24.45)	0.88	-0.27 (-0.44, -0.11)	0.001	-0.19 (-0.44, 0.06)	0.14
PCB-180	-4.57 (-14.58, 5.45)	0.37	23.84 (0.58, 47.11)	0.05	-0.08 (-0.14, -0.01)	0.02	0.12 (-0.14, 0.38)	0.35	-0.05 (-0.14, 0.05)	0.35	-7.05 (-29.46, 15.35)	0.54	-0.29 (-0.45, -0.13)	0.0004	-0.22 (-0.47, 0.03)	0.09
PCB-189	-3.17 (-9.15, 2.81)	0.30	14.54 (0.63, 28.44)	0.04	-0.05 (-0.09, -0.01)	0.01	0.06 (-0.09, 0.22)	0.43	-0.03 (-0.09, 0.03)	0.35	-2.63 (-15.99, 10.73)	0.70	-0.097 (-0.19, -0.001)	0.05	-0.19 (-0.33, -0.04)	0.01
PCB-194	-0.24 (-5.21, 4.74)	0.93	6.61 (-4.96, 18.19)	0.26	-0.02 (-0.05, 0.02)	0.31	0.05 (-0.08, 0.18)	0.41	-0.04 (-0.09, 0.005)	0.08	2.58 (-8.59, 13.75)	0.65	-0.08 (-0.16, 0)	0.05	-0.05 (-0.17, 0.07)	0.43
PCB-206	-3.58 (-12.24, 5.07)	0.42	25.73 (5.67, 45.80)	0.01	-0.07 (-0.13, -0.01)	0.02	-0.03 (-0.26, 0.19)	0.77	-0.09 (-0.17, -0.004)	0.04	-11.44 (-30.71, 7.83)	0.25	-0.23 (-0.37, -0.09)	0.001	-0.27 (-0.48, -0.06)	0.01
PCB-209	-2.75 (-10.58, 5.09)	0.49	17.71 (-0.49, 35.92)	0.06	-0.09 (-0.14, -0.04)	0.0006	0.01 (-0.19, 0.22)	0.91	-0.08 (-0.16, -0.005)	0.04	-8.46 (-25.92, 9.01)	0.34	-0.24 (-0.36, -0.11)	0.0002	-0.26 (-0.45, -0.07)	0.008
OCDD	-7.42 (-13.91, -0.93)	0.03	23.43 (8.35, 38.51)	0.0024	0.003 (-0.04, 0.05)	0.89	0.11 (-0.05, 0.28)	0.18	-0.01 (-0.08, 0.05)	0.69	8.99 (-5.53, 23.52)	0.22	0.05 (-0.06, 0.15)	0.36	-0.02 (-0.18, 0.14)	0.83
HCB	-13.58 (-22.88, -4.28)	0.004	5.91 (-15.77, 27.58)	0.59	-0.014 (-0.08, 0.05)	0.65	0.21 (-0.03, 0.45)	0.09	-0.03 (-0.12, 0.06)	0.51	0.85 (-20.15, 21.85)	0.94	-0.005 (-0.15, 0.15)	0.95	-0.03 (-0.26, 0.20)	0.78
TNC	-5.22 (-11.97, 1.53)	0.13	9.32 (-6.42, 25.06)	0.25	0.025 (-0.02, 0.07)	0.27	-0.04 (-0.22, 0.13)	0.62	-0.002 (-0.07, 0.06)	0.95	-4.89 (-20.25, 10.47)	0.53	-0.04 (-0.15, 0.07)	0.50	-0.02 (-0.19, 0.14)	0.79
p,p'-DDE	5.66 (1.50, 9.81)	0.008	16.68 (7.02, 26.34)	0.0007	0.032 (0.005, 0.06)	0.022	0.002 (-0.11, 0.11)	0.98	-0.01 (-0.05, 0.03)	0.64	4.37 (-4.99, 13.72)	0.36	0.005 (-0.06, 0.07)	0.89	0.16 (0.06, 0.26)	0.003
BDE-47	-1.04 (-6.87, 4.79)	0.73	-0.15 (-13.72, 13.42)	0.98	0.025 (-0.01, 0.06)	0.21	-0.05 (-0.20, 0.10)	0.49	0.004 (-0.05, 0.06)	0.90	-8.43 (-21.41, 4.55)	0.20	-0.03 (-0.13, 0.06)	0.50	-0.084 (-0.23, 0.06)	0.25

β: beta coefficient; CI: confidence interval; ICAM-1: intercellular adhesion molecule 1; VCAM-1: vascular cell adhesion protein 1; IL-6: interleukin 6; TNF-α: tumor necrosis factor α, MCP-1: monocyte chemotactic protein-1; CRP: C-reactive protein; TLC: total leucocyte count; PCB: polychlorinated biphenyls; OCDD: octachlorodibenzo-*p*-dioxin; HCB: hexachlorobenzene; TNC: *trans*-nonachlordane; *p,p*'-DDE: 2,2-bis (4-chlorophenyl)-1,1-dichloroethene; BDE-47: bromodiphenyl ether 47; BDE: brominated diphenyl ether.

Model A- Linear regression model adjusted for sex and kidney function.

Table S2. Association [β (95% CI)] of individual POPs with inflammatory markers in model B.

POP	ICAM-1	p-value	VCAM-1	p-value	E-selectin	p-value	IL-6	p-value	TNF-α	p-value	MCP-1	p-value	CRP	p-value	TLC	p-value
PCB-74	-5.34 (-13.20, 2.52)	0.18	14.44 (-3.89, 32.77)	0.12	-0.004 (-0.06, 0.05)	0.87	0.09 (-0.12, 0.30)	0.41	-0.001 (-0.08, 0.08)	0.98	-6.27 (-24.12, 11.59)	0.49	-0.03 (-0.15, 0.09)	0.61	-0.18 (-0.36, 0.001)	0.05
PCB-99	-1.74 (-8.36, 4.87)	0.61	6.90 (-8.51, 22.31)	0.38	0.002 (-0.04, 0.05)	0.92	0.13 (-0.04, 0.31)	0.14	-0.005 (-0.07, 0.06)	0.89	-14.72 (-29.67, 0.23)	0.05	-0.04 (-0.14, 0.07)	0.51	-0.15 (-0.3, 0)	0.05
PCB-105	-2.52 (-9.38, 4.33)	0.47	9.25 (-6.74, 25.24)	0.26	-0.004 (-0.05, 0.04)	0.87	-0.01 (-0.19, 0.17)	0.92	.005 (-0.06, 0.07)	0.89	-7.85 (-23.39, 7.67)	0.32	-0.04 (-0.15, 0.07)	0.45	-0.17 (-0.33, -0.02)	0.03
PCB-118	-2.20 (-9.81, 5.41)	0.57	15.79 (-1.91, 33.50)	0.08	-0.005 (-0.06, 0.05)	0.84	0.08 (-0.12, 0.29)	0.43	-0.006 (-0.08, 0.07)	0.89	-6.81 (-24.06, 10.44)	0.44	-0.05 (-0.16, 0.07)	0.46	-0.18 (-0.35, -0.004)	0.046
PCB-126	12.26 (7.93, 16.59)	$2.30*10^{-8}$	13.47 (3.26, 23.67)	0.01	0.001 (-0.03, 0.03)	0.92	-0.01 (-0.13, 0.10)	0.82	.011 (-0.03, 0.06)	0.63	-1.06 (-11.28, 9.16)	0.84	.06 (-0.01, 0.13)	0.11	0.12 (0.02, 0.22)	0.017
PCB-138	0.48 (-7.98, 8.93)	0.91	17.03 (-2.65, 36.70)	0.09	-0.007 (-0.06, 0.05)	0.81	0.17 (-0.06, 0.39)	0.15	-0.03 (-0.12, 0.05)	0.45	-8.88 (-28.04, 10.28)	0.36	-0.09 (-0.23, 0.04)	0.16	-0.07 (-0.27, 0.12)	0.47
PCB-153	-0.21 (-9.55, 9.12)	0.96	23.29 (1.58, 45.00)	0.04	-0.003 (-0.06, 0.06)	0.94	0.17 (-0.08, 0.42)	0.18	-0.04 (-0.13, 0.06)	0.42	-9.91 (-31.04, 11.22)	0.36	-0.11 (-0.26, 0.03)	0.13	-0.12 (-0.33, 0.10)	0.28
PCB-156	-9.13 (-18.94, 0.67)	0.07	27.24 (4.4, 50.07)	0.02	-0.02 (-0.09, 0.05)	0.55	0.15 (-0.11, 0.41)	0.27	-0.02 (-0.11, 0.08)	0.75	12.58 (-9.67, 34.83)	0.27	-0.05 (-0.2, 0.11)	0.55	-0.08 (-0.30, 0.14)	0.49
PCB-157	-6.89 (-15.74, 1.96)	0.13	20.04 (-0.56, 40.63)	0.06	-0.038 (-0.10, 0.02)	0.20	0.04 (-0.19, 0.28)	0.72	-0.03 (-0.12, 0.06)	0.52	-6.85 (-26.96, 13.26)	0.51	-0.07 (-0.21, 0.07)	0.33	-0.01 (-0.21, 0.19)	0.92
PCB-169	4.39 (-4.59, 13.37)	0.34	36.87 (16.05, 57.69)	0.0005	-0.04 (-0.10, 0.02)	0.19	0.19 (-0.05, 0.43)	0.12	-0.02 (-0.11, 0.07)	0.65	6.40 (-13.98, 26.77)	0.54	-0.05 (-0.19, 0.09)	0.50	-0.06 (-0.27, 0.14)	0.55
PCB-170	-1.786 (-12.52, 8.95)	0.74	40.42 (15.53, 65.30)	0.002	-0.02 (-0.09, 0.05)	0.65	0.22 (-0.07, 0.50)	0.14	-0.04 (-0.14, 0.07)	0.52	3.33 (-20.99, 27.65)	0.79	-0.07 (-0.23, 0.10)	0.44	-0.12 (-0.37, 0.12)	0.32
PCB-180	-2.7 (-13.37, 7.97)	0.62	40.94 (16.21, 65.66)	0.001	-0.02 (-0.09, 0.05)	0.52	0.17 (-0.11, 0.46)	0.24	-0.05 (-0.16, 0.06)	0.37	-1.20 (-25.36, 22.95)	0.92	-0.07 (-0.23, 0.10)	0.43	-0.12 (-0.37, 0.12)	0.33
PCB-189	-1.163 (-7.48, 5.15)	0.72	27.82 (13.21, 42.43)	0.0002	-0.025 (-0.07, 0.02)	0.23	0.11 (-0.05, 0.28)	0.18	-0.02 (-0.08, 0.05)	0.57	0.15 (-14.12, 14.41)	0.98	.02 (-0.08, 0.12)	0.65	-0.1 (-0.24, 0.04)	0.17
PCB-194	.729 (-4.45, 5.90)	0.78	12.66 (0.63, 24.69)	0.04	0.007 (-0.03, 0.04)	0.67	0.05 (-0.09, 0.19)	0.49	-0.06 (-0.11, -0.004)	0.04	4.80 (-6.97, 16.56)	0.42	.01 (-0.07, 0.09)	0.80	-0.007 (-0.12, 0.11)	0.91
PCB-206	-1.11 (-10.41, 8.18)	0.81	41.36 (19.89, 62.84)	0.0002	-0.01 (-0.07, 0.05)	0.68	-0.02 (-0.27, 0.23)	0.87	-0.10 (-0.19, -0.005)	0.04	-7.28 (-28.25, 13.68)	0.50	-0.02 (-0.17, 0.12)	0.77	-0.17 (-0.38, 0.04)	0.11
PCB-209	.33 (-8.17, 8.83)	0.94	35.87 (16.21, 55.54)	0.0004	-0.04 (-0.09, 0.02)	0.20	0.04 (-0.19, 0.27)	0.73	-0.09 (-0.17, -0.003)	0.04	-0.51 (-19.72, 18.70)	0.96	-0.02 (-0.15, 0.11)	0.75	-0.12 (-0.31, 0.07)	0.23
OCDD	-7.16 (-13.87, -0.46)	0.04	15.92 (0.3, 31.53)	0.05	0.004 (-0.04, 0.05)	0.87	0.09 (-0.09, 0.26)	0.34	-0.03 (-0.10, 0.04)	0.37	16.10 (0.94, 31.25)	0.04	.06 (-0.05, 0.16)	0.29	0.076 (-0.08, 0.23)	0.33
BDE-47	-2.31 (-8.28, 3.66)	0.45	-5.70 (-19.62, 8.23)	0.42	0.02 (-0.02, 0.06)	0.31	-0.05 (-0.21, 0.11)	0.52	.003 (-0.06, 0.06)	0.92	-10.21 (-23.69, 3.26)	0.14	-0.06 (-0.15, 0.04)	0.25	-0.13 (-0.27, 0.001)	0.05

β: beta coefficient; CI: confidence interval; ICAM-1: intercellular adhesion molecule 1; VCAM-1: vascular cell adhesion protein 1; IL-6: interleukin 6; TNF-α: tumor necrosis factor α; MCP-1: monocyte chemotactic protein-1; CRP: C-reactive protein; TLC: total leucocyte count, PCB: polychlorinated biphenyls; OCDD: octachlorodibenzo-*p*-dioxin; BDE: brominated diphenyl ether.

Model B: Linear regression model adjusted for sex, kidney function, smoking, body mass index, waist circumference, blood glucose, systolic blood pressure, high density lipoprotein cholesterol, low density lipoprotein cholesterol, triglycerides, exercise habits and education

Table S3. Association [β (95% CI)] of TEQ and PCB-126 with ICAM-1 into different groups based on median BMI or smoking status[a].

Group	TEQ	p-value	PCB-126	p-value
<Median BMI	11.47 (0.94, 21.99)	0.03	10.5 (4.14, 16.86)	0.001
>=Median BMI	25.08 (13.59, 36.57)	$2.3*10^{-5}$	11.58 (5.34, 17.82)	0.0003
Smokers	10.63 (-23.85, 45.11)	0.55	11.85 (-8.39, 32.09)	0.26
Non-smokers	16.74 (8.89, 24.59)	$3.0*10^{-4}$	9.99 (5.50, 14.49)	$1.5*10^{-5}$

β: beta coefficient; CI: confidence interval; TEQ: toxic equivalency value; PCB: polychlorinated biphenyls; ICAM-1: intercellular adhesion molecule 1.

[a]Linear regression model adjusted for sex, kidney function, smoking, body mass index, waist circumference, blood glucose, systolic blood pressure, high density lipoprotein cholesterol, low density lipoprotein cholesterol, triglycerides, exercise habits and education.

Median BMI = 26.6 kg/m^2.

Table S4. Association [β (95% CI)] of summary measures of POPs and medication with inflammatory markers studied[a].

Marker	TEQ	p-value	Sum of PCBs	p-value	Sum of OC Pest	p-value
**No Asp**						
ICAM-1	16.60 (8.08, 25.11)	$1.5*10^{-4}$	0.27 (-0.46, 0.99)	0.47	0.92 (-2.13, 3.98)	0.55
VCAM-1	30.07 (10.45, 49.70)	0.003	2.59 (0.94, 4.25)	0.002	4.53 (-2.49, 11.56)	0.21
**No Asp or Cort**						
ICAM-1	16.66 (8.022, 25.29)	$2.0*10^{-4}$	0.28 (-0.45, 1.02)	0.45	1.15 (-1.97, 4.26)	0.47
VCAM-1	31.57 (11.68, 51.46)	0.002	2.65 (0.97, 4.33)	0.002	5.68 (-1.47, 12.83)	0.2
**No Asp or Cort or NSAID**						
ICAM-1	15.62 (6.79, 24.44)	$6.0*10^{-4}$	0.217 (-0.53, 0.96)	0.57	1.06 (-2.11, 4.23)	0.51
VCAM-1	29.64 (9.15, 50.12)	0.005	2.52 (0.81, 4.24)	0.004	5.22 (-2.12, 12.56)	0.16

β: beta coefficient; CI: confidence interval; TEQ: toxic equivalency value; PCB: polychlorinated biphenyls; OC: organochlorine; Pest: pesticide; Asp: aspirin; Cort: cortisone; NSAID: non steroid anti-inflammatory drug; ICAM-1: intercellular adhesion molecule 1; VCAM-1: vascular cell adhesion protein 1.

[a]Linear regression model adjusted for sex, kidney function, smoking, body mass index, waist circumference, blood glucose, systolic blood pressure, high density lipoprotein cholesterol, low density lipoprotein cholesterol, triglycerides, exercise habits and education.

Table S5. Association [β (95% CI)] of all the confounders with inflammatory markers studied[a].

Variable	ICAM-1	p-value	VCAM-1	p-value	IL-6	p-value	E-selectin	p-value	MCP-1	p-value	TNF-α	p-value	CRP	p-value	TLC	p-value
Sex	5.54 (-2.21, 13.30)	0.16	-32.86 (-50.78, -14.93)	0.0003	-0.19 (-0.38, 0.01)	0.06	-0.09 (-0.14, -0.04)	0.001	2.36 (-14.76, 19.48)	0.79	-0.06 (-0.14, 0.015)	0.12	0.08 (-0.04, 0.21)	0.19	-0.22 (-0.41, -0.03)	0.02
Smoking	36.64 (24.14, 49.14)	$1.2*10^{-8}$	-20.90 (-50.19, 8.38)	0.16	-0.01 (-0.33, 0.31)	0.94	-0.01 (-0.09, 0.07)	0.81	18.40 (-9.53, 46.32)	0.2	-0.05 (-0.17, 0.07)	0.43	0.16 (-0.04, 0.36)	0.12	1.48 (1.19, 1.77)	$4.6*10^{-23}$
Education	-4.50 (-9.05, 0.06)	0.05	-10.07 (-20.75, 0.61)	0.06	-0.05 (-0.17, 0.07)	0.4	0.001 (-0.03, 0.03)	0.95	-4.67 (-14.77, 5.42)	0.36	-0.007 (-0.05, 0.04)	0.78	-0.06 (-0.14, 0.01)	0.1	-0.14 (-0.25, -0.03)	0.01
Motion	-5.6 (-10.9, -0.3)	0.04	-4.63 (-16.98, 7.71)	0.46	-0.08 (-0.21, 0.06)	0.27	-0.01 (-0.05, 0.02)	0.45	-13.23 (-24.71, -1.75)	0.02	-0.03 (-0.09, 0.02)	0.2	-0.12 (-0.2, -0.03)	0.007	-0.30 (-0.42, -0.17)	$2.9*10^{-6}$
Age	-59.4 (-84.2, -34.7)	$2.8*10^{-6}$	-92.30 (-150.21, -34.34)	0.002	-0.12 (-0.75, 0.52)	0.72	-0.12 (-0.29, 0.04)	0.14	3.79 (-51.67, 59.24)	0.89	-0.001 (-0.25, 0.24)	0.99	0.21 (-0.19, 0.61)	0.3	-0.92 (-1.52, -0.32)	0.003
BMI	1.18 (0.29, 2.07)	0.01	5.74 (3.69, 7.80)	$5.5*10^{-8}$	0.01 (-0.008, .037)	0.22	0.02 (0.01, 0.03)	$4.6*10^{-8}$	1.59 (-0.39, 3.56)	0.12	0.004 (-0.005, 0.01)	0.41	0.06 (0.05, 0.08)	$2.7*10^{-18}$	0.04 (0.02, 0.06)	0.005
Waist	0.43 (0.10, 0.77)	0.01	2.47 (1.71, 3.24)	$3.7*10^{-10}$	0.009 (0, 0.02)	0.04	0.01 (0.007, 0.01)	$8.3*10^{-12}$	0.43 (-0.31, 1.18)	0.26	0.004 (0, 0.007)	0.04	0.02 (0.02, 0.03)	$1.7*10^{-16}$	0.02 (0.01, 0.03)	$6.1*10^{-7}$
Glucose	17.9 (-0.5, 36.3)	0.06	108.11 (65.69, 150.52)	$7.0*10^{-7}$	-0.12 (-0.58, 0.35)	0.62	0.29 (0.17, 0.41)	$2.3*10^{-6}$	13.54 (-27.18, 54.25)	0.51	0.03 (-0.15, 0.21)	0.75	0.57 (0.28, 0.86)	0.0001	1.22 (0.78, 1.66)	$5.8*10^{-8}$
SBP	-0.05 (-0.22, 0.12)	0.59	-0.02 (-0.42, 0.37)	0.91	0.003 (-0.001, 0.007)	0.18	0.002 (0, 0.003)	0.006	-0.16 (-0.54, 0.22)	0.4	0 (-0.002, 0.001)	0.78	0.003 (0.001, 0.006)	0.02	0.001 (-0.01, 0.01)	0.53
HDL	-14.0 (-23.1, -4.9)	0.003	-70.61 (-91.34, -49.89)	$4.9*10^{-11}$	-0.17 (-0.40, 0.06)	0.15	-0.12 (-0.18, -0.06)	0.0001	-6.22 (-26.28, 13.84)	0.54	-0.16 (-0.25, -0.07)	0.0006	-0.49 (-0.64, -0.35)	$2.0*10^{-11}$	-0.75 (-0.96, -0.53)	$1.2*10^{-11}$
LDL	-3.68 (-8.13, 0.76)	0.1	-28.73 (-38.93, -18.54)	$4.3*10^{-8}$	-0.07 (-0.19, 0.04)	0.21	-0.03 (-0.06, -0.002)	0.04	4.75 (-5.06, 14.55)	0.34	-0.07 (-0.11, -0.03)	0.002	0.033 (-0.04, 0.11)	0.36	-0.14 (-0.24, -0.03)	0.01
TG	15.35 (6.26, 24.43)	0.001	27.07 (5.89, 48.24)	0.01	-0.01 (-0.24, 0.22)	0.93	0.17 (0.11, 0.23)	$5.2*10^{-8}$	43.98 (24.06, 63.90)	$2.0*10^{-5}$	0.08 (-0.007, 0.17)	0.07	0.30 (0.16, 0.45)	$4.4*10^{-6}$	0.59 (0.38, 0.81)	$1.1*10^{-7}$
GFR	34.3 (19.2, 49.5)	$1.0-10^{-5}$	-48.12 (-83.58, -12.66)	0.008	-0.15 (-0.54, 0.24)	0.44	-0.03 (-0.13, 0.07)	0.61	7.77 (-25.99, 41.52)	0.65	-0.28 (-0.42, -0.13)	0.0003	-0.46 (-0.70, -0.22)	0.00023	0.08 (-0.29, 0.45)	0.67

β: beta coefficient; CI: confidence interval; ICAM-1: intercellular adhesion molecule 1; VCAM-1: vascular cell adhesion protein 1; IL-6: interleukin 6; MCP-1: monocyte chemotactic protein-1; TNF-α: tumor necrosis factor α; CRP: C-reactive protein; TLC: total leucocyte count; BMI: body mass index; SBP: systolic blood pressure; HDL: high density lipoprotein cholesterol; LDL: low density lipoprotein cholesterol; TG: triglyceride; GFR: glomerular filtration rate.

[a]Univariate analysis.

Table S6. Association [β (95% CI)] of all the confounders with summary measures of POPs, pesticides and dioxin studied[a].

Variable	TEQ	p-value	Sum of OCP	p-value	Sum of PCBs	p-value	OCDD	p-value	HCB	p-value	*p,p*'-DDE	p-value	BDE-47	p-value	TNC	p-value
Sex	-0.13 (-0.19, -0.06)	0.0003	0.29 (0.10, 0.48)	0.003	-0.49 (-1.29, 0.32)	0.24	0.18 (0.10, 0.25)	$2.9*10^{-6}$	0.17 (0.12, 0.22)	$5.0*10^{-10}$	0.10 (-0.02, 0.21)	0.10	-0.18 (-0.26, -0.10)	$2.5*10^{-4}$	-0.17 (-0.25, -0.10)	$2.8*10^{-6}$
Smoking	0.09 (-0.03, 0.20)	0.13	0.09 (-0.22, 0.39)	0.58	0.36 (-0.94, 1.67)	0.59	-0.14 (-0.26, -0.02)	0.03	-0.02 (-0.10, 0.07)	0.71	0.07 (-0.12, 0.26)	0.49	0.01 (-0.13, 0.14)	0.94	-0.01 (-0.13, 0.11)	0.91
Education	-0.01 (-0.05, 0.03)	0.78	0.07 (-0.04, 0.18)	0.22	0.18 (-0.29, 0.66)	0.45	0.01 (-0.04, 0.05)	0.81	0.06 (0.03, 0.09)	0.00036	0.01 (-0.06, 0.08)	0.80	-0.03 (-0.08, 0.02)	0.18	0.04 (-0.01, 0.08)	0.1
Motion	0.007 (-0.04, 0.05)	0.76	-0.08 (-0.21, 0.05)	0.20	0.065 (-0.48, 0.62)	0.82	0.02 (-0.04, 0.07)	0.53	-0.003 (-0.04, 0.03)	0.87	-0.04 (-0.12, 0.04)	0.32	-0.06 (-0.12, -0.01)	0.04	-0.03 (-0.08, 0.02)	0.3
Age	-0.90 (-1.11, -0.69)	$4.5*10^{-16}$	1.03 (0.42, 1.63)	0.0009	-2.06 (-4.65, 0.54)	0.12	0.25 (0.01, 0.49)	0.04	0.72 (0.55, 0.89)	$1.2*10^{-16}$	-0.51 (-0.89, -0.14)	0.01	-0.21 (-0.49, 0.06)	0.12	0.39 (0.16, 0.63)	0.001
BMI	-0.01 (-0.02, -0.01)	0.0003	0.07 (0.05, 0.09)	$1.6*10^{-10}$	-0.16 (-0.25, -0.06)	0.001	0.01 (-0.001, 0.02)	0.08	0.01 (0.002, 0.02)	0.008	0.04 (0.03, 0.05)	$1.0*10^{-8}$	0.003 (-0.01, 0.01)	0.55	0.01 (0.01, 0.02)	0.00085
Waist	-0.004 (-0.01, -0.01)	0.01	0.02 (0.01, 0.03)	$3.8*10^{-7}$	-0.06 (-0.09, -0.02)	0.001	-0.001 (-0.01, 0.01)	0.55	0.001 (-0.01, 0.01)	0.57	0.01 (0.01, 0.02)	$1.3*10^{-6}$	0.003 (-0.01, 0.01)	0.12	0.01 (0.01, 0.01)	$8.2*10^{-7}$
Glucose	0.10 (-0.06, 0.27)	0.21	0.71 (0.26, 1.16)	0.002	0.04 (-1.87, 1.95)	0.96	0.01 (-0.17, 0.19)	0.89	0.07 (-0.05, 0.20)	0.26	0.58 (0.31, 0.86)	$3.3*10^{-5}$	0.12 (-0.08, 0.32)	0.23	0.27 (0.10, 0.45)	0.002
SBP	0 (-0.01, 0.01)	0.78	0.01 (0.01, 0.01)	$3.0*10^{-6}$	0.01 (-0.004, 0.03)	0.14	0.001 (-0.01, 0.01)	0.4	0.001 (0, 0.002)	0.12	0.006 (0.01, 0.01)	$1.0*10^{-4}$	0.001 (-0.01, 0.01)	0.43	0 (-0.01, 0.002)	0.63
HDL	-0.01 (-0.09, 0.07)	0.87	-0.10 (-0.32, 0.12)	0.39	0.72 (-0.23, 1.66)	0.14	0.10 (0.01, 0.19)	0.03	0.10 (0.04, 0.16)	0.002	-0.21 (-0.35, -0.07)	0.0025	-0.13 (-0.23, -0.03)	0.008	-0.10 (-0.19, -0.02)	0.02
LDL	0.05 (0.01, 0.09)	0.02	0.2 (0.10, 0.31)	0.0002	1.09 (0.63, 1.55)	$3.2*10^{-6}$	-0.10 (-0.14, -0.06)	$8.3*10^{-6}$	-0.01 (-0.04, 0.02)	0.57	-0.15 (-0.21, -0.08)	$1.6*10^{-5}$	-0.09 (-0.14, -0.05)	0.0001	-0.04 (-0.08, 0.01)	0.05
TG	0.25 (0.17, 0.33)	$1.0*10^{-9}$	1.09 (0.88, 1.30)	$5.2*10^{-23}$	3.74 (2.83, 4.65)	$3.0*10^{-15}$	-0.22 (-0.31, -0.13)	$1.1*10^{-6}$	-0.06 (-0.12, 0.01)	0.09	0.20 (0.06, 0.33)	0.005	-0.06 (-0.16, 0.04)	0.27	0.15 (0.07, 0.24)	0.0005
GFR	0.40 (0.26, 0.53)	$8.0*10^{-9}$	-0.59 (-0.96, -0.22)	0.002	1.65 (0.07, 3.24)	0.04	-0.16 (-0.31, -0.01)	0.04	-0.42 (-0.52, -0.31)	$9.2*10^{-15}$	0.49 (0.27, 0.72)	$2.5*10^{-5}$	0.07 (-0.10, 0.23)	0.43	-0.37 (-0.51, -0.23)	$3.8*10^{-7}$

β: beta coefficient; CI: confidence interval; TEQ: total equivalency value; OCP: organochlorine pesticide; PCB: polychlorinated biphenyls; OCDD: octachlorodibenzo-*p*-dioxin; HCB: hexachlorobenzene; *p,p*'-DDE: 2,2-bis (4-chlorophenyl)-1,1-dichloroethene; BDE-47: bromodiphenyl ether 47; TNC: *trans*-nonachlordane; BMI: body mass index; SBP: systolic blood pressure; HDL: high density lipoprotein cholesterol; LDL: low density lipoprotein cholesterol; TG: triglyceride; GFR: glomerular filtration rate.

[a]Univariate analysis.

Table S7. Association [β (95% CI)] of all the confounders with PCBs (PCB-74 to PCB-156) studied[a].

Variable	PCB-74	p-value	PCB-99	p-value	PCB-105	p-value	PCB-118	p-value	PCB-126	p-value	PCB-138	p-value	PCB-153	p-value	PCB-156	p-value
Sex	0.19 (0.12, 0.25)	$1.1*10^{-8}$	-0.02 (-0.10, 0.05)	0.54	0.15 (0.07, 0.22)	0.00013	0.17 (0.10, 0.23)	$9.7*10^{-7}$	-0.19 (-0.30, -0.08)	0.001	-0.07 (-0.12, -0.01)	0.03	-0.09 (-0.14, -0.04)	0.0009	-0.11 (-0.16, -0.06)	$1.3*10^{-5}$
Smoking	-0.10 (-0.20, 0.01)	0.07	0.02 (-0.10, 0.14)	0.7	-0.27 (-0.39, -0.15)	$1.0*10^{-5}$	-0.26 (-0.37, -0.15)	$2.8*10^{-6}$	0.10 (-0.09, 0.28)	0.31	0.07 (-0.02, 0.16)	0.15	0.06 (-0.02, 0.15)	0.14	0.08 (-0.003, 0.16)	0.06
Education	0.10 (0.06, 0.14)	$1.3*10^{-7}$	0.03 (-0.02, 0.07)	0.2	0.09 (0.05, 0.13)	$8.0*10^{-4}$	0.07 (0.03, 0.11)	0.00027	0.03 (-0.03, 0.1)	0.34	-0.003 (-0.04, 0.03)	0.87	0.005 (-0.03, 0.04)	0.77	0.02 (-0.02, 0.05)	0.33
Motion	0.02 (-0.03, 0.06)	0.43	-0.02 (-0.07, 0.03)	0.35	0.005 (-0.05, 0.06)	0.85	-0.004 (-0.05, 0.04)	0.87	0.01 (-0.07, 0.09)	0.75	-0.02 (-0.06, 0.02)	0.31	-0.007 (-0.04, 0.03)	0.71	0.01 (-0.02, 0.05)	0.51
Age	0.55 (0.34, 0.75)	$1.6*10^{-7}$	0.19 (-0.05, 0.43)	0.12	0.35 (0.11, 0.59)	0.004	0.36 (0.14, 0.57)	0.001	-1.77 (-2.12, -1.42)	$5.0*10^{-22}$	-0.05 (-0.24, 0.13)	0.58	-0.10 (-0.27, 0.07)	0.27	-0.05 (-0.22, 0.11)	0.53
BMI	0.01 (0.002, 0.02)	0.01	0.01 (0.003, 0.02)	0.009	0.03 (0.02, 0.04)	$4.3*10^{-10}$	0.02 (0.02, 0.03)	$7.0*10^{-9}$	-0.02 (-0.03, -0.002)	0.03	0.003 (-0.003, 0.01)	0.34	-0.007 (-0.01, -0.01)	0.02	-0.02 (-0.03, -0.02)	$3.8*10^{-13}$
Waist	0.001 (-0.01, 0.01)	0.79	0.004 (0, 0.01)	0.03	0.01 (0.005, 0.01)	$2.7*10^{-6}$	0.01 (0.003, 0.01)	0.00012	-0.003 (-0.01, 0.002)	0.19	0.002 (-0.001, 0.01)	0.16	-0.002 (-0.01, 0.01)	0.15	-0.01 (-0.01, -0.01)	$9.0*10^{-9}$
Glucose	0.06 (-0.09, 0.21)	0.45	0.13 (-0.05, 0.30)	0.15	0.25 (0.07, 0.42)	0.006	0.22 (0.06, 0.38)	0.006	0.27 (0.002, 0.54)	0.05	0.14 (0.007, 0.28)	0.04	0.14 (0.01, 0.26)	0.03	0.02 (-0.10, 0.14)	0.73
SBP	0.002 (0.001, 0.01)	0.0008	0.003 (0.01, 0.01)	0.002	0.004 (0.002, 0.01)	$2.3*10^{-5}$	0.003 (0.002, 0.01)	$5.5*10^{-5}$	-0.001 (-0.01, 0.001)	0.42	0.001 (0, 0.003)	0.04	0.001 (-0.01, 0.01)	0.35	-0.001 (-0.002, 0)	0.04
HDL	0.07 (-0.01, 0.15)	0.06	-0.05 (-0.13, 0.04)	0.28	0.04 (-0.05, 0.13)	0.35	0.03 (-0.05, 0.11)	0.47	-0.08 (-0.21, 0.06)	0.25	-0.07 (-0.14, -0.01)	0.05	-0.04 (-0.10, 0.03)	0.26	0.04 (-0.03, 0.10)	0.25
LDL	-0.02 (-0.05, 0.02)	0.35	-0.05 (-0.09, -0.01)	0.03	-0.06 (-0.1, -0.02)	0.008	-0.07 (-0.11, -0.04)	0.0002	-0.10 (-0.16, -0.03)	0.004	-0.06 (-0.10, -0.03)	0.0001	-0.07 (-0.1, -0.04)	$4.8*10^{-6}$	-0.06 (-0.09, -0.03)	0.00016
TG	0.03 (-0.05, 0.10)	0.45	0.11 (0.02, 0.19)	0.01	0.13 (0.05, 0.22)	0.003	0.07 (-0.01, 0.15)	0.09	0.03 (-0.11, 0.16)	0.71	0.042 (-0.03, 0.11)	0.22	-0.02 (-0.08, .05)	0.6	-0.05 (-0.11, 0.01)	0.13
GFR	-0.22 (-0.34, -0.09)	0.0008	-0.03 (-0.17, 0.12)	0.72	-0.16 (-0.31, -0.01)	0.03	-0.16 (-0.30, -0.03)	0.02	0.88 (0.66, 1.10)	$6.3*10^{-15}$	0.04 (-0.07, 0.16)	0.48	0.08 (-0.03, 0.18)	0.15	0.06 (-0.04, 0.16)	0.25

β: beta coefficient; CI: confidence interval; PCB: polychlorinated biphenyls; BMI: body mass index; SBP: systolic blood pressure; HDL: high density lipoprotein cholesterol; LDL: low density lipoprotein cholesterol; TG: triglyceride; GFR: glomerular filtration rate.

[a]Univariate analysis.

Table S8. Association [β (95% CI)] of all the confounders with PCBs (PCB-157 to PCB-209) studied[a].

Variable	PCB-157	p-value	PCB-169	p-value	PCB-170	p-value	PCB-180	p-value	PCB-189	p-value	PCB-194	p-value	PCB-206	p-value	PCB-209	p-value
Sex	-0.11 (-0.17, -0.05)	0.00013	-0.20 (-0.26, -0.14)	$7.1*10^{-12}$	-0.18 (-0.22, -0.13)	$7.0*10^{-13}$	-0.20 (-0.24, -0.15)	$4.0*10^{-15}$	-0.22 (-0.30, -0.14)	$6.5*10^{-8}$	-0.23 (-0.33, -0.13)	$6.4*10^{-6}$	-0.21 (-0.26, -0.15)	$1.4*10^{-12}$	-0.22 (-0.28, -0.16)	$1.8*10^{-11}$
Smoking	0.05 (-0.05, 0.14)	0.33	0.06 (-0.04, 0.15)	0.24	0.09 (0.007, 0.17)	0.03	0.09 (0.01, 0.17)	0.03	-0.04 (-0.17, 0.09)	0.57	0.16 (-0.01, 0.32)	0.06	0.05 (-0.04, 0.14)	0.28	0.07 (-0.03, 0.18)	0.17
Education	0.01 (-0.02, 0.04)	0.57	-0.02 (-0.05, 0.017)	0.33	0.001 (-0.03, 0.03)	0.92	0.005 (-0.02, 0.03)	0.73	-0.01 (-0.06, 0.04)	0.71	0.02 (-0.04, 0.08)	0.55	-0.002 (-0.04, 0.03)	0.91	0.005 (-0.03, 0.04)	0.79
Motion	0.01 (-0.03, 0.05)	0.54	-0.002 (-0.04, 0.04)	0.93	0.01 (-0.02, 0.04)	0.57	0.01 (-0.02, 0.05)	0.41	-0.003 (-0.06, 0.05)	0.92	0.02 (-0.05, 0.09)	0.55	0.02 (-0.02, 0.06)	0.39	0.03 (-0.01, 0.08)	0.16
Age	0.07 (-0.12, 0.25)	0.47	-0.54 (-0.73, -0.36)	$8.0*10^{-9}$	-0.26 (-0.42, -0.11)	0.001	-0.32 (-0.48, -0.16)	0.0001	-0.37 (-0.63, -0.11)	0.006	-1.13 (-1.45, -0.81)	$4.8*10^{-12}$	-0.46 (-0.64, -0.28)	$1.1*10^{-6}$	-0.65 (-0.86, -0.45)	$4.5*10^{-10}$
BMI	-0.02 (-0.03, -0.01)	$1.0*10^{-9}$	-0.02 (-0.03, -0.01)	$2.0*10^{-9}$	-0.02 (-0.03, -0.02)	$1.8*10^{-16}$	-0.03 (-0.031, -0.02)	$1.8*10^{-19}$	-0.03 (-0.04, -0.02)	$4.6*10^{-11}$	-0.05 (-0.06, -0.03)	$1.1*10^{-14}$	-0.03 (-0.04, -0.02)	$1.7*10^{-17}$	-0.04 (-0.04, -0.03)	$1.0*10^{-23}$
Waist	-0.01 (-0.01, -0.003)	$3.5*10^{-6}$	-0.01 (-0.007, -0.002)	0.0003	-0.01 (-0.01, -0.01)	$3.0*10^{-9}$	-0.01 (-0.01, -0.01)	$1.4*10^{-10}$	-0.01 (-0.01, -0.01)	$6.1*10^{-6}$	-0.02 (-0.02, -0.01)	$2.1*10^{-11}$	-0.01 (-0.01, -0.01)	$3.5*10^{-11}$	-0.01 (-0.01, -0.01)	$2.6*10^{-16}$
Glucose	-0.02 (-0.15, 0.12)	0.81	0.13 (-0.01, 0.27)	0.06	0.07 (-0.05, 0.18)	0.27	0.05 (-0.07, 0.16)	0.45	-0.04 (-0.24, 0.15)	0.66	-0.12 (-0.36, 0.12)	0.33	-0.03 (-0.17, 0.10)	0.64	-0.10 (-0.25, 0.06)	0.21
SBP	-0.001 (-0.002, 0)	0.08	-0.002 (-0.003, 0.01)	0.01	-0.001 (-0.002, 0.01)	0.03	-0.001 (-0.003, 0.01)	0.007	-0.001 (-0.003, 0.001)	0.19	-0.002 (-0.004, 0.01)	0.09	-0.001 (-0.003, 0.01)	0.02	-0.002 (-0.003, 0.01)	0.01
HDL	0.06 (-0.01, 0.13)	0.08	-0.01 (-0.08, 0.06)	0.77	0.004 (-0.05, 0.06)	0.9	0.004 (-0.06, 0.06)	0.91	0.03 (-0.07, 0.13)	0.54	0.03 (-0.09, 0.15)	0.61	0.02 (-0.05, 0.08)	0.65	0.05 (-0.02, 0.13)	0.17
LDL	-0.04 (-0.07, -0.01)	0.01	-0.09 (-0.12, -0.06)	$1.4*10^{-7}$	-0.06 (-0.08, -0.03)	$6.6*10^{-5}$	-0.07 (-0.10, -0.05)	$4.5*10^{-7}$	-0.04 (-0.08, 0.01)	0.13	-0.12 (-0.18, -0.06)	0.00006	-0.07 (-0.11, -0.04)	$9.9*10^{-6}$	-0.07 (-0.11, -0.04)	0.0001
TG	-0.08 (-0.15, -0.02)	0.01	-0.07 (-0.14, 0.01)	0.05	-0.06 (-0.12, -0.004)	0.04	-0.11 (-0.16, -0.05)	0.0004	-0.15 (-0.25, -0.05)	0.002	-0.17 (-0.29, -0.05)	0.005	-0.11 (-0.18, -0.04)	0.002	-0.16 (-0.24, -0.09)	$3.0*10^{-5}$
GFR	0.09 (-0.02, 0.20)	0.11	0.33 (0.21, 0.44)	$1.8*10^{-8}$	0.18 (0.09, 0.28)	0.0001	0.21 (0.11, 0.31)	$3.0*10^{-5}$	0.18 (0.02, 0.34)	0.03	0.87 (0.67, 1.06)	$4.4*10^{-18}$	0.33 (0.22, 0.45)	$8.0*10^{-9}$	0.52 (0.40, 0.65)	$2.7*10^{-16}$

β: beta coefficient; CI: confidence interval; PCB: polychlorinated biphenyls; BMI: body mass index; SBP: systolic blood pressure; HDL: high density lipoprotein cholesterol; LDL: low density lipoprotein cholesterol; TG: triglyceride; GFR: glomerular filtration rate.

[a]Univariate analysis.
